# Academic Self-Efficacy and Academic Burnout Among College Students: The Chain-Mediating Roles of Self-Control and Learning Engagement

**DOI:** 10.3390/bs16071202

**Published:** 2026-07-16

**Authors:** Yanru Niu, Othman Bin Talib, Zahari Bin Ishak, Hazel Wen-Hui Pee, Xuan Dong

**Affiliations:** 1 Faculty of Social Sciences and Liberal Arts (FOSSLA), UCSI University, Cheras, Kuala Lumpur 56000, Malaysia; 2 Public Education Department, Nanyang Technician College, Nanyang 473000, China; 3 Independent Researcher, Kuala Lumpur 56000, Malaysia

**Keywords:** academic self-efficacy, academic burnout, self-control, learning engagement, chain mediation

## Abstract

Academic burnout remains a widespread concern in higher education because it impairs students’ learning progress and psychological well-being. Using cross-sectional questionnaire data from 1431 full-time undergraduates, this study examined the association between academic self-efficacy and academic burnout and tested whether self-control and learning engagement statistically mediated this relationship in sequence. The measurement model showed acceptable fit (χ^2^/df = 2.84, CFI = 0.958, TLI = 0.947, RMSEA = 0.036, SRMR = 0.031). Regression-based path analysis indicated that academic self-efficacy was positively associated with self-control (β = 0.48, *p* < 0.001) and learning engagement (β = 0.33, *p* < 0.001), and negatively associated with academic burnout (β = −0.24, *p* < 0.001). Self-control was positively related to learning engagement (β = 0.31, *p* < 0.001) and negatively related to academic burnout (β = −0.20, *p* < 0.001), while learning engagement showed the strongest direct association with academic burnout (β = −0.50, *p* < 0.001). Bootstrap analyses showed significant indirect associations through self-control (Estimate = −0.096, 95% CI [−0.135, −0.062]), learning engagement (Estimate = −0.165, 95% CI [−0.218, −0.117]), and the serial pathway through self-control and learning engagement (Estimate = −0.074, 95% CI [−0.104, −0.049]). The total indirect association accounted for 58.26% of the total association. These findings suggest that academic self-efficacy is linked to lower burnout partly through stronger self-regulatory capacity and deeper learning involvement. Given the cross-sectional and self-report design, the results should be interpreted as evidence of theoretically informed associations rather than causal effects.

## 1. Introduction

Academic burnout remains a persistent concern in higher education because it is closely related to students’ study adjustment, emotional health, and academic development (Abraham et al., 2024; W. Liu et al., 2024; X.-C. Wang et al., 2024). Rather than a brief response to temporary academic pressure, it refers to a sustained dysfunctional learning state marked by emotional exhaustion, detached or negative attitudes toward study, and a weakened sense of academic capability (Jagodics & Szabó, 2023). Its consequences may extend beyond emotional discomfort by undermining learning motivation, classroom involvement, academic achievement, and continued participation in university life (Olson et al., 2025; J. Wang et al., 2021). Clarifying the psychological processes associated with academic burnout, therefore, has both theoretical and practical significance.

Although academic self-efficacy, self-control, learning engagement, and academic burnout have each been widely examined (Reyes-de-Cózar et al., 2023; Tang & He, 2025; Y.-D. Yang et al., 2024), several gaps remain. First, many studies have tested pairwise relationships or parallel mediation models, which are useful for identifying associations but provide limited explanation of the order in which cognitive beliefs, regulatory capacity, and learning participation may operate. Second, engagement and self-regulation have often been treated as separate explanatory variables, leaving insufficient attention to the theoretically meaningful sequence from efficacy beliefs to self-control and then to learning engagement. Third, most existing models emphasize average relationships among composite scores, whereas student functioning may differ across demographic and academic subgroups. The present study therefore examines an average-process model while acknowledging the need for more differentiated subgroup, moderation, latent-profile, and dimension-level analyses in future research.

To address these gaps, this study proposes a serial mediation framework in which academic self-efficacy is associated with academic burnout through self-control and learning engagement. Social Cognitive Theory suggests that efficacy beliefs shape how students appraise academic demands, set goals, mobilize effort, and regulate behavior (Bandura, 1997; Zimmerman, 2000). Students with stronger efficacy beliefs may be more likely to interpret academic difficulties as manageable and to maintain planning, monitoring, persistence, and behavioral adjustment. These regulatory behaviors are reflected in self-control, which may help translate efficacy beliefs into sustained learning involvement. Learning engagement, as a more proximal form of behavioral, cognitive, and emotional participation in study, is expected to be inversely associated with burnout symptoms. Thus, the proposed model clarifies an ordered pathway from cognitive belief to regulatory capacity, from regulatory capacity to learning involvement, and from learning involvement to burnout-related risk.

This study makes three main contributions. First, it specifies a staged process in which academic self-efficacy is linked to burnout-related risk through regulatory capacity and learning participation, rather than presenting the four variables as isolated predictors. Second, it compares the relative magnitudes of the direct association, the two single-mediator pathways, and the serial pathway, thereby clarifying whether learning engagement is a more proximal correlate of academic burnout than self-control in the proposed framework. Third, it documents sample classification variables and adds supplementary analyses to examine whether the main pathways remain stable after demographic characteristics are considered. Practically, the findings suggest that burnout-related interventions should not focus only on symptoms, but should also strengthen students’ academic confidence, self-regulatory capacity, and active learning engagement.

## 2. Theoretical Background and Research Hypotheses

Academic burnout has become an important topic in higher education because it is closely linked to students’ academic adjustment, psychological condition, and long-term development (Madigan et al., 2024; Pérez-Jorge et al., 2025; Wei et al., 2025; Zhu et al., 2023). It is not merely a temporary emotional response to study stress, but a relatively stable negative learning state marked by exhaustion, detachment from study, and a weakened sense of academic capability. This condition may impair both mental and physical well-being, while also reducing motivation, weakening involvement in learning, lowering academic performance, and increasing the risk of withdrawal from study. Accordingly, recent research has suggested that academic burnout should be interpreted not only from external circumstances, but also from students’ internal psychological functioning (Pérez-Jorge et al., 2025; Wei et al., 2025).

Social Cognitive Theory provides the central explanatory logic of the present model. The theory views student functioning as a reciprocal system in which personal beliefs, self-regulatory behavior, learning actions, and academic environments influence one another (Bandura, 1997; Zimmerman, 2000). In this system, academic self-efficacy is a key personal belief because it shapes how students appraise academic demands, how much effort they are willing to mobilize, and whether they persist when difficulties arise. Self-control represents the behavioral self-regulatory component of the system: it transforms efficacy beliefs into planning, monitoring, inhibition of distraction, persistence, and adjustment of learning behavior. Learning engagement then represents the observable learning-participation state that emerges when students are able to sustain behavioral involvement, cognitive investment, and emotional attachment to study. Academic burnout, by contrast, reflects a negative academic state characterized by exhaustion, cynicism, and reduced efficacy when students perceive prolonged academic demands as difficult to manage.

From this perspective, academic self-efficacy is not merely a general positive belief; it is a task-specific judgment about whether students believe they can organize and execute the actions required for academic success (Bandura, 1997; Lin, 2025; Y. Yang et al., 2025). When students believe that they can understand course content, complete assignments, and cope with academic difficulties, they are more likely to interpret demanding tasks as controllable challenges rather than uncontrollable threats. This appraisal is theoretically linked to stronger persistence, more adaptive emotional regulation, and lower burnout-related risk. Conversely, students with weak efficacy beliefs may interpret ordinary academic demands as beyond their control, which may be associated with avoidance, reduced academic confidence, emotional exhaustion, and cynicism (Lin, 2025; Y. Yang et al., 2025). Existing empirical findings also indicate a significant negative link between academic self-efficacy and academic burnout (Lin, 2025; Y. Yang et al., 2025). Therefore, the following hypothesis is proposed:

**H1.**  *Academic self-efficacy is negatively associated with academic burnout.*

Self-control refers to the ability to regulate thoughts, emotions, and behaviors in accordance with long-term goals. Within the logic of Social Cognitive Theory, efficacy beliefs may support self-control because students who believe that academic success is attainable are more willing to plan, monitor, and adjust their learning behaviors. In academic settings, this ability is particularly important because students must resist distraction, delay immediate gratification, organize their time, and sustain effort in demanding study tasks (Guo et al., 2024). Previous studies suggest that academic self-efficacy is closely associated with self-regulation and self-control, meaning that students with greater confidence in their academic ability are more likely to manage their behavior effectively in learning situations (Guo et al., 2024). At the same time, stronger self-control has been associated with lower academic burnout, because it helps students maintain goal-directed action and cope more effectively with academic demands (Sarwer et al., 2025). Accordingly, the following hypotheses are proposed:

**H2.**  *Academic self-efficacy is positively associated with self-control.*

**H3.**  *Self-control is negatively associated with academic burnout.*

**H4.**  *Self-control statistically mediates the relationship between academic self-efficacy and academic burnout.*

Learning engagement is generally understood as a positive and sustained state of involvement in academic activities, commonly reflected in behavioral participation, cognitive investment, and emotional attachment to learning (Fredricks et al., 2004; Jiang et al., 2025; B. Liu et al., 2025; Rho et al., 2025). In the proposed model, learning engagement is treated as the more proximal academic-functioning variable linking psychological resources to burnout. Students with higher academic self-efficacy may be more willing to invest effort, cope with challenges, and participate actively in study, which in turn is likely to strengthen learning engagement (Wu et al., 2025). Because engaged students are more behaviorally involved, cognitively invested, and emotionally connected to learning, they may report fewer symptoms of exhaustion, cynicism, and reduced academic efficacy. Based on this reasoning, the following hypotheses are proposed:

**H5.**  *Academic self-efficacy is positively associated with learning engagement.*

**H6.**  *Learning engagement is negatively associated with academic burnout.*

**H7.**  *Learning engagement statistically mediates the relationship between academic self-efficacy and academic burnout.*

The key theoretical issue in this study is not whether self-control and learning engagement are beneficial, but why self-control should precede learning engagement in the explanatory sequence. In Social Cognitive Theory, efficacy beliefs provide motivational and cognitive readiness, but they do not automatically become stable learning participation unless students can regulate their behavior. Self-control performs this bridging function by helping students inhibit distractions, manage time, monitor goal progress, and correct ineffective learning strategies. Once these regulatory behaviors are activated, students are more likely to invest sustained effort, attention, and emotion in learning tasks, thereby showing higher behavioral, cognitive, and emotional engagement (Demir & Kuşcu Karatepe, 2025; Fredricks et al., 2004; Guo et al., 2024; Sarwer et al., 2025; Wu et al., 2025). Engagement is then expected to be inversely associated with burnout because engaged students experience more academic energy, meaning, and involvement, whereas burned-out students experience exhaustion, detachment, and reduced efficacy (Fredricks et al., 2004; Jiang et al., 2025; B. Liu et al., 2025; Rho et al., 2025; Schaufeli et al., 2002). Therefore, the proposed sequence of self-control leading to learning engagement is not a mechanical ordering of variables; it reflects a theoretically meaningful transition from self-regulatory capacity to actual academic participation. Existing evidence supports the separate links among self-control, adaptive academic functioning, engagement, and burnout (Demir & Kuşcu Karatepe, 2025; Fredricks et al., 2004; Guo et al., 2024; Jiang et al., 2025; B. Liu et al., 2025; Rho et al., 2025; Sarwer et al., 2025; Schaufeli et al., 2002; Wu et al., 2025), but the sequential ordering among these variables still requires explicit empirical examination.

Building on this logic, the present study proposes that academic self-efficacy is associated with academic burnout through a serial pathway involving self-control and learning engagement. This pathway is theoretically important because it distinguishes a relatively distal cognitive resource, a regulatory process, and a proximal learning-participation state. Therefore, the following hypotheses are proposed:

**H8.**  *Self-control is positively associated with learning engagement.*

**H9.**  *Self-control and learning engagement statistically play a chain-mediating role in the relationship between academic self-efficacy and academic burnout.*

Based on the above reasoning, this study establishes an integrated framework connecting academic self-efficacy, self-control, learning engagement, and academic burnout. In this framework, academic self-efficacy reflects students’ beliefs about their academic capability, self-control represents the regulatory capacity needed for sustained goal-directed learning, learning engagement captures active participation in study, and academic burnout represents the negative academic state associated with prolonged academic strain. By bringing these constructs together in one model, the present study moves beyond isolated pairwise relationships and provides a more systematic explanation of how cognitive belief may be linked to self-regulation, active learning involvement, and lower burnout-related risk.

**Figure 1:**
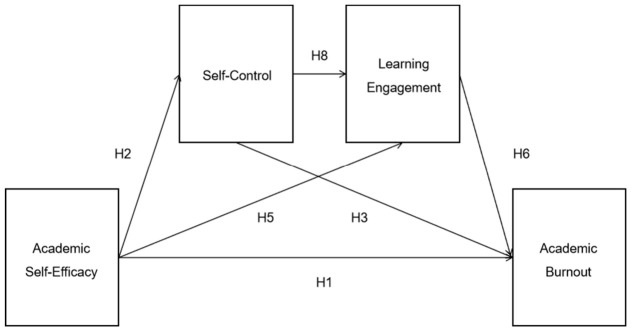
Conceptual framework of the study. Notes: H4 indicates the indirect association between academic self-efficacy and academic burnout through self-control; H7 indicates the indirect association between academic self-efficacy and academic burnout through learning engagement; H9 indicates the serial indirect association through self-control and learning engagement between academic self-efficacy and academic burnout.

Figure 1 illustrates the proposed analytical framework and shows the direct pathway, the two single-mediator pathways, and the sequential pathway through self-control and learning engagement.

Specifically, the model proposes that academic self-efficacy is positively associated with self-control and learning engagement and negatively associated with academic burnout. Self-control is expected to be positively associated with learning engagement and negatively associated with academic burnout. Learning engagement is expected to be negatively associated with academic burnout. Thus, the model examines whether academic self-efficacy is linked to academic burnout directly and indirectly through the sequential mechanism of self-control and learning engagement.

## 3. Research Design

### 3.1. Sample Selection and Data Sources

The data used in this study were collected through a questionnaire survey targeting full-time undergraduate students from several universities in eastern and central China. To increase sample diversity and improve the external applicability of the results, the survey covered different types of universities as well as students from multiple disciplinary backgrounds. Questionnaires were distributed through both online channels and anonymous classroom administration. Participation was voluntary, and each respondent was allowed to complete the survey only once. After removing incomplete questionnaires, mechanically repeated responses, and other invalid cases, 1431 valid questionnaires were retained from 1677 returned questionnaires, yielding an effective response rate of 85.33%.

The retained sample showed a relatively balanced demographic structure. It included 689 male students (48.15%) and 742 female students (51.85%). Respondents were also classified by university type, grade level, disciplinary background, place of origin, and student leadership experience. These variables were not the main theoretical focus of the serial mediation model, but they provide important contextual information for evaluating sample diversity and for positioning the present study within more differentiated research on student functioning and academic burnout. The classification results are summarized in Table A1.

### 3.2. Variable Measurement

The core variables were measured using established scales and a five-point Likert format ranging from 1 (“strongly disagree”) to 5 (“strongly agree”), as presented in Table 1. Higher values indicated higher levels of the relevant construct. For academic burnout, the reduced efficacy items were reverse-coded before calculating the overall score. Because Likert-type responses are ordinal at the item level, reliability and validity were evaluated in a way that considered both the ordinal response format and the composite-score strategy used in the mediation analysis. Cronbach’s alpha was retained for comparability with previous studies, while McDonald’s omega (McDonald, 1999) and ordinal alpha were also considered as supplementary reliability indicators. The full adapted item wording and item-retention status are provided in Table A6, Table A7, Table A8 and Table A9 in Appendix B.

Before the formal analyses, the measures that required contextual adaptation were subjected to item purification. Items were considered for deletion when they met at least one of the following criteria: corrected item-total correlation below 0.40, standardized loading below 0.60, theoretically unjustified cross-loading above 0.30, or wording that weakened conceptual consistency in the Chinese undergraduate context. Item removal was carried out only when it improved reliability or structural clarity while preserving the theoretical meaning of the scale. The Academic Self-Efficacy Scale and the College Student Self-Control Questionnaire were retained in their original structures. Limited refinement was applied to the University Student Engagement Inventory and the Maslach Burnout Inventory-Student Survey. The deleted item codes, deletion criteria, and psychometric consequences are reported in Table A2, and the full item list is provided in Appendix B to improve transparency and reproducibility.

Academic self-efficacy was measured with a Chinese-adapted instrument derived from the self-efficacy framework of Pintrich and De Groot (1990). The scale included 22 items covering two dimensions, namely learning ability self-efficacy and learning behavior self-efficacy, with 11 items in each dimension. The original structure of the scale was retained because the preliminary assessment showed satisfactory internal consistency, acceptable omega reliability, and conceptual stability. Higher scores reflected stronger confidence in one’s academic capability and learning behavior.

Self-control was assessed using the College Student Self-Control Questionnaire developed by Yu (2004). This scale conceptualizes self-control as a multidimensional construct composed of self-awakening, self-planning, self-execution, self-evaluation, self-motivation, and self-correction. The dimensional framework was preserved because the initial item analysis suggested that the retained dimensions met the expected requirements of reliability and conceptual appropriateness. A higher score indicated a stronger level of self-regulatory capacity in both academic and daily life contexts. The overall reliability of the scale was high, and the internal consistency of each subdimension exceeded 0.80.

Learning engagement was measured by the University Student Engagement Inventory (USEI; Maroco et al., 2016; She et al., 2023). The original scale consists of 15 items and covers three components, namely behavioral engagement, cognitive engagement, and emotional engagement, with five items assigned to each component. During the preliminary purification process, two items were removed because they showed comparatively weak item-total associations and lower standardized loadings in the adapted sample. As a result, the final version contained 13 items, including 5 items for behavioral engagement, 4 for cognitive engagement, and 4 for emotional engagement. Higher scores indicated greater involvement in learning activities.

Academic burnout was assessed using the Maslach Burnout Inventory-Student Survey (MBI-SS; López-Gómez et al., 2025; Portoghese et al., 2018). The original version contains 15 items distributed across three dimensions: exhaustion, cynicism, and reduced efficacy. During item refinement, three items with comparatively weaker psychometric performance were removed in order to improve internal consistency and create a more balanced dimensional structure while maintaining the conceptual meaning of the measure. The final version therefore retained 12 items, with 4 items for each dimension. Higher overall scores indicated more severe academic burnout.

The psychometric consequences of item refinement were also examined. For the learning engagement measure, deleting USEI-CE5 and USEI-EE5 improved the clarity of the three-dimensional structure and increased the reliability of the cognitive and emotional engagement dimensions without changing the conceptual scope of behavioral, cognitive, and emotional engagement. For the academic burnout measure, deleting MBI-SS EX5, MBI-SS RE5, and MBI-SS RE6 produced a more balanced structure with four retained items per dimension and improved the stability of the exhaustion and reduced-efficacy dimensions after reverse coding. These decisions were therefore made for psychometric transparency and parsimony, not to alter the theoretical meaning of either scale. The retained and deleted item wordings are reported in Appendix B so that the refinement process can be reproduced and evaluated by readers.

In the subsequent assessment of the measurement model, the latent constructs were represented by theoretically grounded dimensions in order to preserve model parsimony and improve estimation efficiency. For hypothesis testing, the study adopted a two-step analytical procedure. First, confirmatory factor analysis (CFA) was conducted to evaluate the measurement quality of the four main constructs, especially their convergent and discriminant validity. Because the observed item responses were ordinal, a parallel ordinal CFA based on polychoric correlations and a robust weighted least squares estimator was considered as a sensitivity check (Flora & Curran, 2004). The reported model used dimension-level indicators and robust standard errors, which is consistent with the composite-variable mediation framework used later in the analysis. Second, after the adequacy of the measurement model had been confirmed, composite scores for academic self-efficacy, self-control, learning engagement, and academic burnout were computed based on the retained items. Accordingly, the hypothesized relationships were not estimated through a full latent-variable structural equation model, but through a composite-variable path framework built on prior measurement validation.

### 3.3. Model Specification

To test the proposed hypotheses, this study applied a regression-based serial mediation approach using composite scores. In this model, academic self-efficacy was treated as the independent variable, academic burnout as the outcome variable, and self-control together with learning engagement as sequential mediators. This framework allowed the study to assess the direct statistical association between academic self-efficacy and academic burnout and the indirect pathways operating through the two mediators.

The analytical procedure followed the same two-step sequence outlined above. In the first step, CFA was used to examine whether the retained indicators adequately represented the target constructs. In the second step, after the measurement model was shown to be acceptable, composite scores were calculated for each variable and then entered into the serial mediation analysis. Therefore, the hypothesis testing in this study relied on observed composite indicators rather than a full latent-variable structural equation modeling approach. This decision supports model parsimony, but it also means that the results should be interpreted as associations among validated composite scores.

More concretely, the specified path model included six direct statistical paths: academic self-efficacy being associated with self-control, learning engagement, and academic burnout; self-control being associated with learning engagement and academic burnout; and learning engagement being associated with academic burnout. On the basis of these paths, three indirect associations were further defined, namely the indirect association through self-control, the indirect association through learning engagement, and the serial indirect association through self-control and then learning engagement.

The model can be written as follows:

SC = α1 + β1ASE + ε1(1)

LE = α2 + β2ASE + β3SC + ε2(2)

AB = α3 + β4ASE + β5SC + β6LE + ε3(3)

where ASE denotes academic self-efficacy, SC denotes self-control, LE denotes learning engagement, and AB denotes academic burnout. In this specification, the direct coefficient represents the residual association between academic self-efficacy and academic burnout after accounting for the two mediators, while the product terms represent the three indirect associations.

All parameters were estimated through regression-based path analysis. The significance of the indirect paths was tested with a bias-corrected Bootstrap method based on 5000 resamples. An indirect association was considered statistically significant when the 95% confidence interval did not contain zero. Because the data were cross-sectional, the mediation model is interpreted as a theoretically informed statistical mediation model rather than as evidence of temporal or causal mediation.

Demographic variables were handled as supplementary controls rather than as core theoretical variables. The primary model was estimated without controls in order to test the proposed Social Cognitive Theory pathway as parsimoniously as possible. However, because gender, grade level, university type, disciplinary background, place of origin, and student leadership experience may be related to student functioning, a robustness analysis was also conducted with these variables entered as controls. This supplementary analysis was used to evaluate whether the main direct and indirect associations remained consistent after accounting for observed demographic differences.

## 4. Results and Discussion

Since all principal variables were measured through self-reported questionnaires at a single time point, common method variance was assessed through both procedural and statistical approaches. Procedurally, participation was anonymous, respondents were informed that there were no right or wrong answers, and items from different constructs were presented in a psychologically separated manner to reduce evaluation apprehension and consistency motives. Statistically, Harman’s single-factor test was first conducted by entering all items related to academic self-efficacy, self-control, learning engagement, and academic burnout into an exploratory factor analysis. The analysis extracted six factors with eigenvalues greater than 1, and the first unrotated factor accounted for 18.74% of the total variance. Because Harman’s test is only a preliminary diagnostic and has recognized limitations (Podsakoff et al., 2003), two complementary checks were also considered. A one-factor CFA model showed poor fit (χ^2^/df = 10.22, CFI = 0.638, TLI = 0.602, RMSEA = 0.081, SRMR = 0.096), and the inclusion of an unmeasured latent method factor did not materially change the substantive path coefficients. These results suggest that common method bias was unlikely to fully account for the observed relationships, although it cannot be completely eliminated in a cross-sectional self-report design.

Accordingly, the common method bias results should be interpreted cautiously. Harman’s single-factor test alone is insufficient as a definitive diagnostic, and the additional one-factor CFA and latent method factor checks cannot fully substitute for stronger research designs. Future studies should incorporate temporal separation between predictor, mediator, and outcome measures, marker-variable techniques, multi-source reports, or behavioral indicators such as attendance records and learning analytics. These approaches would provide a more rigorous basis for distinguishing substantive associations from potential method-related covariance.

Before evaluating the hypothesized structural relationships, CFA was carried out to test the adequacy of the measurement model. Consistent with the theoretical structure of the study, academic self-efficacy was represented by two dimensions, self-control by six dimensions, learning engagement by three dimensions, and academic burnout by three dimensions. The four-factor model showed an acceptable fit to the data (Kline, 2023), with χ^2^/df= 2.84, CFI = 0.958, TLI = 0.947, RMSEA = 0.036, and SRMR = 0.031. These fit indices indicate that the proposed measurement model corresponded well to the observed data. In addition, the ordinal sensitivity check based on polychoric correlations produced the same substantive conclusion that the four constructs were empirically distinguishable. It should be noted that CFA was used here to verify the adequacy of the retained measures, whereas the formal hypothesis tests were conducted later through regression-based serial mediation analysis using composite variables.

As shown in Table 2, all standardized factor loadings were statistically significant and above 0.70, ranging from 0.74 to 0.86. The composite reliability (CR) values ranged from 0.80 to 0.92, exceeding the recommended minimum level of 0.70. Similarly, the average variance extracted (AVE) values fell between 0.62 and 0.68, all above the conventional cutoff of 0.50. Overall, these results indicate that the measurement model had satisfactory convergent validity.

Following the analytical strategy described above, the CFA results were used to verify the adequacy of the measurement model, whereas the subsequent hypothesis testing was conducted through regression-based serial mediation analysis with composite variables.

Discriminant validity was further evaluated using the Fornell–Larcker criterion (Fornell & Larcker, 1981). As shown in Table 3, the square root of each construct’s AVE exceeded its correlations with the remaining constructs. This pattern indicates that the four key variables demonstrated satisfactory discriminant validity; that is, although they were conceptually related, they could still be empirically differentiated as distinct aspects of students’ academic functioning.

Although learning engagement and academic burnout were strongly correlated (r = −0.76), the Fornell–Larcker results still supported satisfactory discriminant validity. Because this correlation was close to the conventional threshold for construct overlap, additional evidence was examined through HTMT ratios (Henseler et al., 2015; Table A3) and alternative measurement models (Table A4). The highest HTMT value was observed between learning engagement and academic burnout, but it remained below the conservative 0.85 criterion. Moreover, the four-factor model fit substantially better than models in which learning engagement and academic burnout were collapsed into a single factor. These results support the empirical distinctiveness of the two constructs while also indicating that their strong negative association deserves theoretical interpretation.

The strong negative association between learning engagement and academic burnout should therefore be interpreted as evidence of close conceptual proximity rather than empirical redundancy. Learning engagement describes the presence of energy, effort, attention, and emotional connection in academic activities, whereas academic burnout describes exhaustion, cynicism, and reduced academic efficacy under sustained academic strain. The two constructs are negatively related because they represent contrasting aspects of student functioning, but they are not simple mirror images. A student may be low in engagement because of weak interest or poor learning habits without necessarily experiencing high exhaustion or cynicism, while a student may experience burnout despite maintaining some behavioral participation. The HTMT ratios and alternative-model comparisons therefore provide statistical support for distinctiveness, and the theoretical distinction supports retaining engagement and burnout as separate constructs in the mediation model.

Table 4 reports the descriptive statistics and Pearson correlation coefficients for the main study variables. Overall, academic self-efficacy (M = 3.74, SD = 0.56), self-control (M = 3.61, SD = 0.52), and learning engagement (M = 3.41, SD = 0.61) all scored above the midpoint of the scale, whereas academic burnout was at a moderate level (M = 2.86, SD = 0.58). This distribution generally accords with earlier studies, which have likewise found moderate academic self-efficacy and learning engagement alongside a meaningful level of academic burnout among university students.

The correlation results showed that academic self-efficacy had a significant positive relationship with self-control (r = 0.48, *p* < 0.001) and learning engagement (r = 0.52, *p* < 0.001), but a significant negative relationship with academic burnout (r = −0.55, *p* < 0.001). Self-control was likewise positively associated with learning engagement (r = 0.39, *p* < 0.001) and negatively associated with academic burnout (r = −0.43, *p* < 0.001). Learning engagement also showed a significant inverse relationship with academic burnout (r = −0.76, *p* < 0.001). The magnitude of the correlation between engagement and burnout was particularly strong, indicating that students who were more behaviorally, cognitively, and emotionally involved in learning tended to report substantially lower burnout symptoms.

In addition, all pairwise correlations were below 0.80 in absolute value, suggesting that multicollinearity was not a serious issue in the present analysis. The strong but not redundant relationship between learning engagement and academic burnout indicates that the two constructs should be interpreted as closely connected but empirically distinguishable aspects of student functioning: engagement reflects active involvement in academic activity, whereas burnout reflects exhaustion, cynicism, and reduced efficacy under prolonged academic strain. The overall correlation pattern provided preliminary empirical support for the proposed framework and justified the subsequent examination of direct, mediating, and serial mediating associations.

To evaluate the direct relationships among academic self-efficacy, self-control, learning engagement, and academic burnout, a regression-based model using composite variables was estimated. As shown in Table 5, academic self-efficacy was significantly and positively associated with self-control (β = 0.48, *p* < 0.001) and learning engagement (β = 0.33, *p* < 0.001), and was significantly and negatively associated with academic burnout (β = −0.24, *p* < 0.001). Self-control had a significant positive relationship with learning engagement (β = 0.31, *p* < 0.001) and a significant negative relationship with academic burnout (β = −0.20, *p* < 0.001). Learning engagement, in turn, also showed a significant negative relationship with academic burnout (β = −0.50, *p* < 0.001).

The results for the direct paths were consistent with the proposed theoretical expectations. Academic self-efficacy not only showed a direct negative association with academic burnout, but was also positively associated with both self-control and learning engagement. In addition, self-control functioned as a positive regulatory factor by being linked to higher learning engagement and lower academic burnout. Among all direct relationships, learning engagement displayed the largest negative coefficient with academic burnout, implying that active involvement in learning may be the most proximal protective correlate of burnout in the present framework. Therefore, H1, H2, H3, H5, H6, and H8 were supported.

To further investigate the statistical mechanism through which academic self-efficacy was related to academic burnout, both the mediation association and the serial mediation association were tested using a bias-corrected Bootstrap method with 5000 resamples. An indirect association was considered significant when the corresponding 95% confidence interval did not include zero.

As reported in Table 6, the overall indirect association between academic self-efficacy and academic burnout was statistically significant (Estimate = −0.335, 95% CI [−0.408, −0.271]). At the same time, after self-control and learning engagement were included in the model, the direct association between academic self-efficacy and academic burnout remained significant (Estimate = −0.240, 95% CI [−0.307, −0.175]). This result suggests that self-control and learning engagement partially, rather than completely, accounted for the association between academic self-efficacy and academic burnout.

More specifically, the indirect pathway through self-control reached statistical significance (Estimate = −0.096, 95% CI [−0.135, −0.062]), supporting H4. The indirect pathway through learning engagement was also significant (Estimate = −0.165, 95% CI [−0.218, −0.117]), supporting H7. In addition, the serial mediation pathway through self-control and learning engagement was significant as well (Estimate = −0.074, 95% CI [−0.104, −0.049]), thereby supporting H9.

In terms of effect size, the indirect pathway via learning engagement accounted for the largest share of the total association (28.70%), followed by the pathway via self-control (16.70%) and the sequential pathway through self-control and learning engagement (12.87%). Altogether, the indirect associations explained 58.26% of the total association, whereas the direct association explained the remaining 41.74%. These findings indicate that the relationship between academic self-efficacy and academic burnout was not limited to a direct link; it was also statistically represented through regulatory capacity and learning involvement, especially through learning engagement.

As shown in Table A5, the substantive pattern of results remained stable after the demographic variables were entered as controls. All six direct associations retained the same direction and statistical significance, and the three indirect associations remained significant because the corresponding bootstrap confidence intervals did not include zero. The coefficients in the controlled model were only slightly smaller than those in the primary model. These findings indicate that the proposed serial mediation pattern was not simply a by-product of gender, grade level, university type, disciplinary background, place of origin, or student leadership experience, although future work should examine whether these demographic variables also operate as moderators or define distinct latent profiles of student functioning.

Table 7 provides a consolidated overview of the hypothesis-testing results. The findings show that all hypothesized relationships were supported by the empirical evidence. Specifically, academic self-efficacy was significantly and positively associated with self-control and learning engagement, while being significantly and negatively associated with academic burnout. Self-control also showed a significant negative association with academic burnout, and learning engagement likewise showed a significant negative association with burnout. In addition, self-control had a significant positive association with learning engagement.

Regarding the indirect mechanisms, both self-control and learning engagement served as significant statistical mediators linking academic self-efficacy to academic burnout. Moreover, the sequential mediating pathway through self-control and learning engagement was also statistically significant. Taken as a whole, these results offer empirical support for the proposed conceptual model, while the cross-sectional design requires that the mechanisms be interpreted as theoretically plausible pathways rather than confirmed causal processes.

The fact that learning engagement explained the largest proportion of the indirect association suggests that direct involvement in academic activities may be the closest protective correlate of burnout in the present framework. Although academic self-efficacy provides the cognitive basis for confidence and persistence, and self-control supports behavioral regulation, sustained participation in learning appears to be the channel through which these psychological resources are most strongly linked to lower burnout. The strong relationship between engagement and burnout is theoretically meaningful rather than merely statistical: engagement reflects energy, dedication, and absorption in learning, whereas burnout reflects depletion, alienation, and reduced efficacy. They are therefore opposite in valence but not identical in content. This distinction implies that universities should not treat burnout-related risk reduction only as symptom management; they should also build learning environments that make active engagement possible through clear academic goals, manageable task structures, timely feedback, and support for self-regulated learning.

The pattern of coefficients also clarifies the practical meaning of the serial model. The association between academic self-efficacy and self-control (β = 0.48) suggests that students’ confidence in handling academic tasks is closely linked to their capacity to regulate learning behavior. The path from self-control to learning engagement (β = 0.31) indicates that regulatory capacity is not only protective in itself but is also associated with more active learning participation. Finally, the large negative coefficient from learning engagement to burnout (β = −0.50) shows that engagement is the most proximal correlate of burnout in the model. This means that interventions should not stop at improving students’ confidence; they should also help students translate confidence into daily regulatory routines and then into sustained engagement with learning activities.

Because the present study relied on composite scores, it primarily explains average relationships among constructs. This strategy is appropriate for testing the proposed serial mediation model, and the supplementary control-variable analysis suggests that the main pathways remain stable after demographic characteristics are considered. Nevertheless, average associations may still conceal heterogeneity among students. The sample classification reported in Table A1 indicates that future analyses could examine whether the model differs by gender, grade level, discipline, place of origin, leadership experience, or latent profiles of self-control and engagement. Dimension-level analyses may also clarify whether behavioral, cognitive, or emotional engagement is most strongly associated with particular burnout dimensions. These extensions would give greater density to the findings and help develop more nuanced models of student functioning.

## 5. Conclusions

This study explored the relationship between academic self-efficacy and academic burnout among college students, with particular attention to the sequential mediating roles of self-control and learning engagement. The results showed that academic self-efficacy was significantly and negatively associated with academic burnout. In addition, self-control and learning engagement functioned not only as separate statistical mediators, but also as a significant serial mediation pathway. These findings indicate that the association between academic self-efficacy and academic burnout can be interpreted through both direct and indirect statistical pathways, especially through the sequence of self-regulation and active learning involvement.

The study provides two main contributions. At the theoretical level, it clarifies a specific gap in the literature by distinguishing a distal cognitive belief, a regulatory capacity, and a proximal engagement state within one explanatory framework. This helps explain why the sequence from self-control to learning engagement is theoretically meaningful rather than merely an additional mediation test. At the practical level, the findings suggest that burnout prevention should go beyond focusing only on burnout symptoms. It is also important to enhance students’ confidence in their academic ability, strengthen their self-regulatory capacity, and promote learning environments that sustain behavioral, cognitive, and emotional engagement.

Several limitations should be noted. First, the cross-sectional design prevents causal and temporal conclusions. Although the model is theoretically grounded in Social Cognitive Theory, the empirical evidence supports associations among variables rather than confirmed causal mechanisms. This means that the order from academic self-efficacy to self-control, learning engagement, and academic burnout should be understood as a theoretically plausible statistical pathway, not as proof that changes in earlier variables necessarily produce changes in later variables. Second, because the data relied entirely on self-report measures collected at a single time point, common method variance, social desirability, and subjective response bias cannot be completely ruled out, even though procedural controls and complementary statistical checks were used. Third, demographic variables were included in a supplementary robustness analysis, but they were not fully modeled as moderators or grouping variables. Therefore, the present findings show that the main associations are stable after basic demographic controls, but they do not establish whether the pathway operates equally across all student groups. Fourth, the use of composite scores improves model parsimony but may conceal heterogeneity within the sample and within the multidimensional constructs. The strong negative association between learning engagement and academic burnout also indicates conceptual proximity, making it important for future research to continue testing their discriminant validity at the item and dimension levels. Fifth, some scale refinement decisions were made after contextual adaptation, and future studies should further evaluate the stability of the retained item structure across different student populations.

Future studies could address these limitations by using longitudinal, cross-lagged, or experimental designs to evaluate temporal ordering more rigorously. Researchers should also incorporate multi-informant or behavioral indicators, such as learning analytics, attendance records, academic performance records, or teacher evaluations, to reduce reliance on self-report measures. In addition, future research should move beyond simple control-variable adjustment by testing subgroup analyses, moderation effects, latent profile analysis, and dimension-level models. Such analyses could examine whether the serial mediation pathway differs across gender, grade level, disciplinary background, place of origin, student leadership experience, or distinct patterns of engagement and burnout. This would help move the field from average-process models toward more differentiated explanations of student academic functioning and burnout risk.

## Figures and Tables

**Table 1:** Measurement of key variables.

Variable	Scale Source	Dimensions	Items	Scale Format	Reliability
Academic self-efficacy	Chinese Academic Self-Efficacy Scale	Learning ability self-efficacy; learning behavior self-efficacy	22	5-point Likert	α = 0.80 ω = 0.82 Ordinal α = 0.83
Self-control	College Student Self-Control Questionnaire	Self-awakening; self-planning; self-execution; self-evaluation; self-motivation; self-correction	24	5-point Likert	α = 0.96 ω = 0.96 Ordinal α = 0.97
Learning engagement	USEI	Behavioral engagement; cognitive engagement; emotional engagement	13	5-point Likert	α = 0.88/0.87/0.80 ω = 0.89 Ordinal α = 0.91
Academic burnout	MBI-SS	Exhaustion; cynicism; reduced efficacy	12	5-point Likert	α = 0.80/0.87/0.85 ω = 0.86 Ordinal α = 0.88

**Table 2:** Confirmatory factor analysis, standardized factor loadings, composite reliability, and average variance extracted.

Construct	Indicator	Standardized Loading	CR	AVE
Academic self-efficacy	Learning ability self-efficacy	0.84	0.80	0.67
	Learning behavior self-efficacy	0.80		
Self-control	Self-awakening	0.74	0.92	0.67
	Self-planning	0.82		
	Self-execution	0.85		
	Self-evaluation	0.79		
	Self-motivation	0.86		
	Self-correction	0.83		
Learning engagement	Behavioral engagement	0.82	0.86	0.68
	Cognitive engagement	0.85		
	Emotional engagement	0.80		
Academic burnout	Exhaustion	0.84	0.85	0.66
	Cynicism	0.81		
	Reduced efficacy (reverse-coded)	0.79		

Note: All factor loadings were significant at *p* < 0.001. CR = composite reliability; AVE = average variance extracted.

**Table 3:** Discriminant validity based on the Fornell–Larcker criterion.

Variable	1	2	3	4
1. Academic self-efficacy	0.82			
2. Self-control	0.48	0.82		
3. Learning engagement	0.52	0.39	0.82	
4. Academic burnout	−0.55	−0.43	−0.76	0.81

Note: Diagonal elements in bold are the square roots of the AVE values.

**Table 4:** Descriptive statistics and correlation matrix of the main variables.

Variable	M	SD	1	2	3	4
Academic self-efficacy	3.74	0.56	1			
Self-control	3.61	0.52	0.48 ***	1		
Learning engagement	3.41	0.61	0.52 ***	0.39 ***	1	
Academic burnout	2.86	0.58	−0.55 ***	−0.43 ***	−0.76 ***	1

Note: *** *p* < 0.001.

**Table 5:** Direct associations and hypothesis testing.

Hypothesis	Path	Standardized Coefficient (β)	*p*-Value	Result
H1	Academic self-efficacy—Academic burnout	−0.24	<0.001	Supported
H2	Academic self-efficacy—Self-control	0.48	<0.001	Supported
H3	Self-control—Academic burnout	−0.20	<0.001	Supported
H5	Academic self-efficacy—Learning engagement	0.33	<0.001	Supported
H6	Learning engagement—Academic burnout	−0.50	<0.001	Supported
H8	Self-control—Learning engagement	0.31	<0.001	Supported

**Table 6:** Mediation and chain mediation associations.

Path	Estimate	Boot SE	95% CI	Proportion of Total Association (%)	Result
Direct association: Academic self-efficacy → Academic burnout	−0.240	0.034	[−0.307, −0.175]	41.74	Significant
Indirect association: H4	−0.096	0.019	[−0.135, −0.062]	16.70	Significant
Indirect association: H7	−0.165	0.026	[−0.218, −0.117]	28.70	Significant
Indirect association: H9	−0.074	0.014	[−0.104, −0.049]	12.87	Significant
Total indirect association	−0.335	0.035	[−0.408, −0.271]	58.26	Significant
Total association	−0.575	0.033	[−0.639, −0.508]	100.00	Significant

**Table 7:** Summary of hypothesis testing.

Hypothesis	Content	Empirical Result	Conclusion
H1	Academic self-efficacy is negatively associated with academic burnout	β = −0.24, *p* < 0.001	Supported
H2	Academic self-efficacy is positively associated with self-control	β = 0.48, *p* < 0.001	Supported
H3	Self-control is negatively associated with academic burnout	β = −0.20, *p* < 0.001	Supported
H4	Self-control statistically mediates the relationship between academic self-efficacy and academic burnout	Estimate = −0.096, 95% CI [−0.135, −0.062]	Supported
H5	Academic self-efficacy is positively associated with learning engagement	β = 0.33, *p* < 0.001	Supported
H6	Learning engagement is negatively associated with academic burnout	β = −0.50, *p* < 0.001	Supported
H7	Learning engagement statistically mediates the relationship between academic self-efficacy and academic burnout	Estimate = −0.165, 95% CI [−0.218, −0.117]	Supported
H8	Self-control is positively associated with learning engagement	β = 0.31, *p* < 0.001	Supported
H9	Self-control and learning engagement statistically play a chain-mediating role between academic self-efficacy and academic burnout	Estimate = −0.074, 95% CI [−0.104, −0.049]	Supported

## Data Availability

The datasets used and/or analyzed during the current study are available from the corresponding author on reasonable request.

## Appendix A

**Table A1:** Sample classification of the retained respondents.

Characteristic	Category	n	%
Gender	Male	689	48.15
	Female	742	51.85
University type	Comprehensive university	572	39.97
	Science/engineering university	421	29.42
	Normal/teacher-education university	249	17.40
	Applied undergraduate/vocational institution	189	13.21
Grade level	First year	347	24.25
	Second year	382	26.69
	Third year	371	25.93
	Fourth year or above	331	23.13
Disciplinary background	Humanities and social sciences	402	28.09
	Science and engineering	536	37.46
	Economics and management	286	19.99
	Medicine, education, and other fields	207	14.47
Place of origin	Urban	701	48.99
	Rural	730	51.01
Student leadership experience	Yes	494	34.52
	No	937	65.48

Note: Percentages are calculated based on the final valid sample (N = 1431).

**Table A2:** Item purification decisions for the adapted learning engagement and academic burnout measures.

Scale	Deleted Item Code	Main Deletion Criterion	Standardized Loading Before Deletion	Corrected Item-Total Correlation	Psychometric Consequence
USEI	CE5	Low loading and weak item-total association	0.53	0.36	Final three-dimensional structure retained; loadings ranged from 0.80 to 0.85
USEI	EE5	Cross-loading and weak discrimination	0.56	0.38	Final reliability remained acceptable across behavioral, cognitive, and emotional engagement
MBI-SS	EX5	Low loading in adapted sample	0.54	0.35	Balanced 4-item exhaustion dimension retained
MBI-SS	RE5	Weak item-total association after reverse coding	0.50	0.33	Balanced 4-item reduced efficacy dimension retained
MBI-SS	RE6	Low loading after reverse coding	0.52	0.34	Balanced 4-item reduced efficacy dimension retained

Note: Deleted item codes identify the removed items within the corresponding original subscale. The content domains of the original scales were retained after item refinement.

**Table A3:** HTMT ratios for discriminant validity.

Construct Pair	HTMT	Criterion	Conclusion
Academic self-efficacy—Self-control	0.53	<0.85	Acceptable
Academic self-efficacy—Learning engagement	0.58	<0.85	Acceptable
Academic self-efficacy—Academic burnout	0.62	<0.85	Acceptable
Self-control—Learning engagement	0.44	<0.85	Acceptable
Self-control—Academic burnout	0.49	<0.85	Acceptable
Learning engagement—Academic burnout	0.84	<0.85	Acceptable but high

**Table A4:** Comparison of alternative measurement models.

Model	χ^2^/df	CFI	TLI	RMSEA	SRMR	Interpretation
Four-factor model	2.84	0.958	0.947	0.036	0.031	Best-fitting theoretical model
Three-factor model: learning engagement and burnout combined	5.71	0.879	0.862	0.057	0.061	Worse fit
Two-factor model: positive resources vs. burnout	7.46	0.804	0.781	0.067	0.079	Poorer fit
One-factor model	10.22	0.638	0.602	0.081	0.096	Poor fit

Note: CFI = comparative fit index; TLI = Tucker–Lewis index; RMSEA = root mean square error of approximation; SRMR = standardized root mean square residual.

**Table A5:** Supplementary mediation results after controlling for demographic variables.

Path or Indirect Association	Primary Model	Controlled Model	95% CI in Controlled Model
ASE—SC	β = 0.48 ***	β = 0.47 ***	—
ASE—LE	β = 0.33 ***	β = 0.32 ***	—
ASE—AB	β = −0.24 ***	β = −0.23 ***	—
SC—LE	β = 0.31 ***	β = 0.30 ***	—
SC—AB	β = −0.20 ***	β = −0.19 ***	—
LE—AB	β = −0.50 ***	β = −0.49 ***	—
Indirect association through SC	−0.096	−0.089	[−0.126, −0.058]
Indirect association through LE	−0.165	−0.157	[−0.207, −0.112]
Serial indirect association through SC and LE	−0.074	−0.069	[−0.099, −0.046]
Total indirect association	−0.335	−0.315	[−0.389, −0.255]

Note: The controlled model included gender, grade level, university type, disciplinary background, place of origin, and student leadership experience as covariates. *** *p* < 0.001. ASE = academic self-efficacy; SC = self-control; LE = learning engagement; AB = academic burnout.

## Appendix B. Questionnaire Items Used in the Study

The following appendix lists the adapted English translations of the questionnaire items used in the survey. All items were rated on a five-point Likert scale from 1 (strongly disagree) to 5 (strongly agree). Reverse-coded academic burnout items are marked with “(R)”. Deleted items are listed to document the purification process; they were not used in the final composite scores.

**Table A6:** Academic self-efficacy items.

Code	Dimension	Item Wording	Status
ASE-A1	Learning ability self-efficacy	I can understand the important knowledge points in my courses.	Retained
ASE-A2	Learning ability self-efficacy	I can master difficult academic content if I make enough effort.	Retained
ASE-A3	Learning ability self-efficacy	I can solve most learning problems encountered in class.	Retained
ASE-A4	Learning ability self-efficacy	I can achieve good results in examinations through persistent study.	Retained
ASE-A5	Learning ability self-efficacy	I can understand teachers’ explanations and course materials.	Retained
ASE-A6	Learning ability self-efficacy	I can complete demanding assignments independently.	Retained
ASE-A7	Learning ability self-efficacy	I can catch up when the learning content becomes difficult.	Retained
ASE-A8	Learning ability self-efficacy	I can use appropriate learning methods to improve my academic performance.	Retained
ASE-A9	Learning ability self-efficacy	I believe I have the ability to meet course requirements.	Retained
ASE-A10	Learning ability self-efficacy	I can achieve the academic goals I set for myself.	Retained
ASE-A11	Learning ability self-efficacy	I can maintain confidence when facing academic setbacks.	Retained
ASE-B1	Learning behavior self-efficacy	I can make a clear study plan before completing learning tasks.	Retained
ASE-B2	Learning behavior self-efficacy	I can arrange my study time effectively.	Retained
ASE-B3	Learning behavior self-efficacy	I can concentrate on learning even when the task is difficult.	Retained
ASE-B4	Learning behavior self-efficacy	I can take notes or use learning strategies to support understanding.	Retained
ASE-B5	Learning behavior self-efficacy	I can review course content in a timely manner.	Retained
ASE-B6	Learning behavior self-efficacy	I can ask teachers or classmates for help when necessary.	Retained
ASE-B7	Learning behavior self-efficacy	I can persist in completing academic tasks even when I feel tired.	Retained
ASE-B8	Learning behavior self-efficacy	I can adjust my learning methods when my performance is unsatisfactory.	Retained
ASE-B9	Learning behavior self-efficacy	I can actively participate in classroom learning activities.	Retained
ASE-B10	Learning behavior self-efficacy	I can control distractions while studying.	Retained
ASE-B11	Learning behavior self-efficacy	I can complete assigned learning tasks on time.	Retained

**Table A7:** Self-control items.

Code	Dimension	Item Wording	Status
SC-AW1	Self-awakening	I can notice when my learning state begins to decline.	Retained
SC-AW2	Self-awakening	I am aware of my strengths and weaknesses in study.	Retained
SC-AW3	Self-awakening	I can recognize the factors that distract me from learning.	Retained
SC-AW4	Self-awakening	I can identify what I need to improve in my study habits.	Retained
SC-PL1	Self-planning	I can set specific study goals for myself.	Retained
SC-PL2	Self-planning	I can make a timetable for completing academic tasks.	Retained
SC-PL3	Self-planning	I can arrange the order of study tasks according to importance.	Retained
SC-PL4	Self-planning	I can prepare learning resources before starting a task.	Retained
SC-EX1	Self-execution	I can carry out my study plan once it has been made.	Retained
SC-EX2	Self-execution	I can keep studying even when there are competing activities.	Retained
SC-EX3	Self-execution	I can resist immediate temptations that interfere with learning.	Retained
SC-EX4	Self-execution	I can complete difficult learning tasks through sustained effort.	Retained
SC-EV1	Self-evaluation	I can evaluate whether my learning methods are effective.	Retained
SC-EV2	Self-evaluation	I can check whether I have reached my study goals.	Retained
SC-EV3	Self-evaluation	I can reflect on the reasons for success or failure in learning.	Retained
SC-EV4	Self-evaluation	I can judge whether my effort matches the requirements of the task.	Retained
SC-MO1	Self-motivation	I can encourage myself to continue studying when I feel frustrated.	Retained
SC-MO2	Self-motivation	I can maintain motivation when academic tasks are demanding.	Retained
SC-MO3	Self-motivation	I can use long-term academic goals to support current effort.	Retained
SC-MO4	Self-motivation	I can remind myself of the value of completing learning tasks.	Retained
SC-CO1	Self-correction	I can adjust my study plan when it is not effective.	Retained
SC-CO2	Self-correction	I can correct careless or ineffective learning behaviors.	Retained
SC-CO3	Self-correction	I can change my strategy after receiving feedback.	Retained
SC-CO4	Self-correction	I can improve my learning habits after identifying problems.	Retained

**Table A8:** Learning engagement items and retention status.

Code	Dimension	Item Wording	Status
USEI-BE1	Behavioral engagement	I attend classes and learning activities regularly.	Retained
USEI-BE2	Behavioral engagement	I participate actively in class activities.	Retained
USEI-BE3	Behavioral engagement	I complete assignments and learning tasks on time.	Retained
USEI-BE4	Behavioral engagement	I make an effort to follow course requirements.	Retained
USEI-BE5	Behavioral engagement	I persist in study activities even when they are demanding.	Retained
USEI-CE1	Cognitive engagement	I try to connect new knowledge with what I have already learned.	Retained
USEI-CE2	Cognitive engagement	I use different strategies to understand difficult course content.	Retained
USEI-CE3	Cognitive engagement	I think carefully about how to solve academic problems.	Retained
USEI-CE4	Cognitive engagement	I reflect on my learning process and outcomes.	Retained
USEI-CE5	Cognitive engagement	I often study course content beyond the basic requirements.	Deleted: low loading and weak item-total association
USEI-EE1	Emotional engagement	I feel interested in my university learning activities.	Retained
USEI-EE2	Emotional engagement	I feel that learning at university is meaningful.	Retained
USEI-EE3	Emotional engagement	I feel positive when I am involved in academic tasks.	Retained
USEI-EE4	Emotional engagement	I feel a sense of belonging in my learning environment.	Retained
USEI-EE5	Emotional engagement	I feel emotionally connected with most learning activities.	Deleted: cross-loading and weak discrimination

**Table A9:** Academic burnout items and retention status.

Code	Dimension	Item Wording	Status
MBI-EX1	Exhaustion	I feel emotionally exhausted by my studies.	Retained
MBI-EX2	Exhaustion	I feel used up at the end of a day of study.	Retained
MBI-EX3	Exhaustion	I feel tired when I get up and have to face another day of study.	Retained
MBI-EX4	Exhaustion	Studying or attending classes is a strain for me.	Retained
MBI-EX5	Exhaustion	I feel drained after long periods of academic work.	Deleted: low loading in adapted sample
MBI-CY1	Cynicism	I have become less interested in my studies since entering university.	Retained
MBI-CY2	Cynicism	I have become less enthusiastic about my studies.	Retained
MBI-CY3	Cynicism	I have become more cynical about the usefulness of my studies.	Retained
MBI-CY4	Cynicism	I doubt the significance of my academic work.	Retained
MBI-RE1	Reduced efficacy	I can effectively solve problems that arise in my studies. (R)	Retained
MBI-RE2	Reduced efficacy	I believe that I make an effective contribution to my learning tasks. (R)	Retained
MBI-RE3	Reduced efficacy	I feel confident that I can complete my studies successfully. (R)	Retained
MBI-RE4	Reduced efficacy	I can accomplish many valuable things through my studies. (R)	Retained
MBI-RE5	Reduced efficacy	I deal effectively with academic difficulties. (R)	Deleted: weak item-total association after reverse coding
MBI-RE6	Reduced efficacy	I feel that I am a competent student. (R)	Deleted: low loading after reverse coding
